# Priming of pop-out in the spatial-cueing paradigm

**DOI:** 10.3758/s13414-024-02998-0

**Published:** 2024-12-23

**Authors:** Dirk Kerzel, Dominique Lamy

**Affiliations:** 1 Department of Psychology, University of Geneva, 40 Boulevard du Pont d’Arve, CH-1205 Geneva, Switzerland; 2 School of Psychological Sciences, Tel Aviv, University, Tel Aviv, Israel

**Keywords:** Visual search, Priming of pop-out, Attentional priority, Contingent capture

## Abstract

Searching for a unique target is faster when its unique feature repeats than when it changes. The standard account for this priming-of-popout (PoP) phenomenon is that selecting a target increases the attentional priority of its features in subsequent searches. However, empirical tests of this priority account have yielded contradictory findings. Here, we attempted to clarify why support of its predictions has been elusive in spatial-cueing studies. We noted that the repetition manipulation is stronger in PoP studies than in spatial-cueing studies: The target and nontarget features either repeat or swap in the former, whereas in the latter, the target color either repeats or changes while the nontarget color remains constant. Here, we implemented the stronger repetition manipulation in the spatial-cueing paradigm. If PoP affects attentional priority, the cue-validity effect should be larger when the current-cue color and previous-target color match than when they mismatch. The results of Experiment 1 revealed the opposite effect. Moreover, they showed that well-replicated contingent-capture effects do not hold when target and nontarget colors are allowed to swap—an observation that was confirmed in Experiment 2. In Experiment 3, we verified that with our experimental set-up, a subtle manipulation of attentional priority could modulate cue-validity effects. While the present study does not resolve why evidence for the priority account is inconsistent with the spatial-cueing paradigm, they eliminate one possible reason for such inconsistency. They also reveal that allowing the target and distractors color to swap masks contingent-capture effects, thereby highlighting the complexity of spatial-cueing effects.

**Supplementary information:**

The online version contains supplementary material available at 10.3758/s13414-024-02998-0.

## Introduction

How we search our environment at any given moment is greatly influenced by the searches we recently performed. In a seminal demonstration of this phenomenon, Maljkovic and Nakayama (1994) had participants search for a target defined by its unique color: The target was unpredictably either the red diamond among green diamonds or vice versa, and the task was to report whether the left or right side of the target was chipped. Participants responded considerably faster when the target color happened to repeat from the previous trial than when it changed, even though the specific color of the target had no relevance for the task. Such better search performance when the target-defining feature repeats relative to when it does not is a well replicated finding referred to as feature intertrial priming or priming-of-popout (PoP; Maljkovic & Nakayama, 1994).

The canonical account for this phenomenon is that selecting a target with a certain feature increases the attentional priority of that feature in subsequent searches (Anderson, 2021; Awh et al., 2012; Luck et al., 2021; Maljkovic & Nakayama, 1994; Wolfe, 2021). This initial account was primarily supported by an early study showing that observers make fewer erroneous saccades when the target-defining feature repeats from the previous trial than when it changes (McPeek et al., 1999). This priority account of PoP was later challenged by an episodic-retrieval account suggesting that PoP reflects mechanisms that occur after the target is found and therefore does not influence attentional priority. The main piece of evidence for this episodic-retrieval account is that PoP interacts with response repetition (e.g., Huang et al., 2004): There is a response-repetition benefit on successive trials when the target and nontarget features also repeat and a response-repetition cost when these features swap. However, later studies demonstrated that this response-related influence on visual search can be dissociated from an earlier, perceptual influence (e.g., Lamy et al., 2010): For instance, the benefit of repeating the target-defining feature is observed even when the response changes on successive trials and therefore, it cannot reflect only retrieval of the previous response when the target feature repeats (e.g., Huang et al., 2004; Lamy & Yashar, 2011; Lamy et al., 2010). Still, while this residual benefit may indicate that PoP influences attentional priority, it could instead indicate that PoP speeds processes that occur after the target is found and before response-related processes are initiated (e.g., deciding whether the attended object is indeed the target).

In order to clarify the role of PoP on attentional priority, Ramgir and Lamy (2022) recently reviewed the studies that are specifically suited to testing the priority account. They found that the evidence across different measures of attentional priority is inconsistent. In particular, they showed that studies relying on the spatial-cueing paradigm have yielded mixed results. In a typical spatial-cueing experiment (e.g., Folk et al., 1992), participants look for a target and shortly before the search display is presented, a cue (typically a stimulus salient by its unique feature—for instance, a red object among white ones) appears at one of the potential target locations. Faster search performance when the target appears at the same location as the cue (valid-cue trials) than at a different location (invalid-cue trials) is taken to indicate that the cue captured attention. This paradigm has been pivotal in demonstrating the prominent role of observers’ goals in guiding attention. Specifically, many studies have shown contingent capture (e.g., Folk & Remington, 1998): A cue produces a validity effect only when it matches the target-defining feature—for example, in search for a green target, when the cue is green but not when it is red (for a review, see Büsel et al., 2020).

Several authors noted that because the target-matching cue always shares the previous target color, whereas the nonmatching cue never does, selecting the target on the previous trial may increase the priority of target-matching cue on the current trial, and intertrial priming (i.e., PoP) rather than goal-directed attention, might therefore account for the contingent-capture pattern of results (e.g., Belopolsky et al., 2010; Folk & Remington, 2008). However, the studies that tested this possibility have yielded inconsistent findings: while some reported that a cue produces a larger cue-validity effect when it shares the previously selected target feature than when it does not (e.g., Belopolsky et al., 2010; Folk & Remington, 2008), other studies found no such effect (Biderman et al., 2017; Eimer & Kiss, 2010; Yashar et al., 2017; see Ramgir & Lamy, 2022, for review).

The present study was an attempt to resolve the contradiction between studies showing that intertrial priming affects attentional priority using other measures (see Ramgir & Lamy, 2022, for review) and the mixed picture that arises from spatial-cueing studies. Our starting point was the observation that in the original paradigm pioneered by Maljkovic and Nakayama (1994), as well as in many studies supporting the idea that PoP affects attentional priority (e.g., Burnham, 2023; Hickey et al., 2011; Leonard & Egeth, 2008; McPeek et al., 2000; Wirth et al., 2023), the effect was defined as the benefit of repeating both the target and nontarget colors relative to when these were swapped. As a result, the PoP effect reflected the sum of the benefit of repeating the target color, the benefit of repeating the nontarget color, the cost of having the target take on the previous nontarget color, and the cost of having the nontargets take on the previous target color (Bichot & Schall, 1999; Kristjánsson & Driver, 2008; Lamy & Yashar, 2008; Maljkovic & Nakayama, 1994, for demonstrations of these distinct effects). By contrast, in most of the relevant spatial-cueing studies, the nontarget color remained constant across trials, with PoP reflecting only the benefit of repeating the target color. Therefore, it may not be surprising that PoP was weaker in these studies and that its influence on the cue-validity effect may have been more difficult to detect.

To test this possibility, in Experiment 1 of the present study, participants searched for a target defined by its unique color (red among green nontargets or vice versa, unpredictably), and the cue could also be either red or green. Thus, the target and nontargets colors either repeated or swapped, allowing us to measure the sequential effects of both target and nontarget colors. As participants had to search for the uniquely colored object, their attentional set was tuned to searching for any color singleton (singleton-detection mode; Bacon & Egeth, 1994). We therefore expected the magnitude of the cue-validity effect to be similar whether the current cue color matched or did not match the current target color (henceforth, cue-target match vs. mismatch; e.g., Anderson & Folk, 2010; Folk & Remington, 2008; Irons et al., 2012). The crucial comparison for the present purposes concerned the match between the colors of the current cue and the previous target. If selecting a target among nontargets increases the priority of objects sharing the target color and decreases the priority of objects sharing the nontarget color, the cue-validity effect should be larger when the current cue color matches the previous target color than when it matches the previous nontarget color (henceforth, target-cue match vs. mismatch).

## Experiment 1

**Fig. 1:**
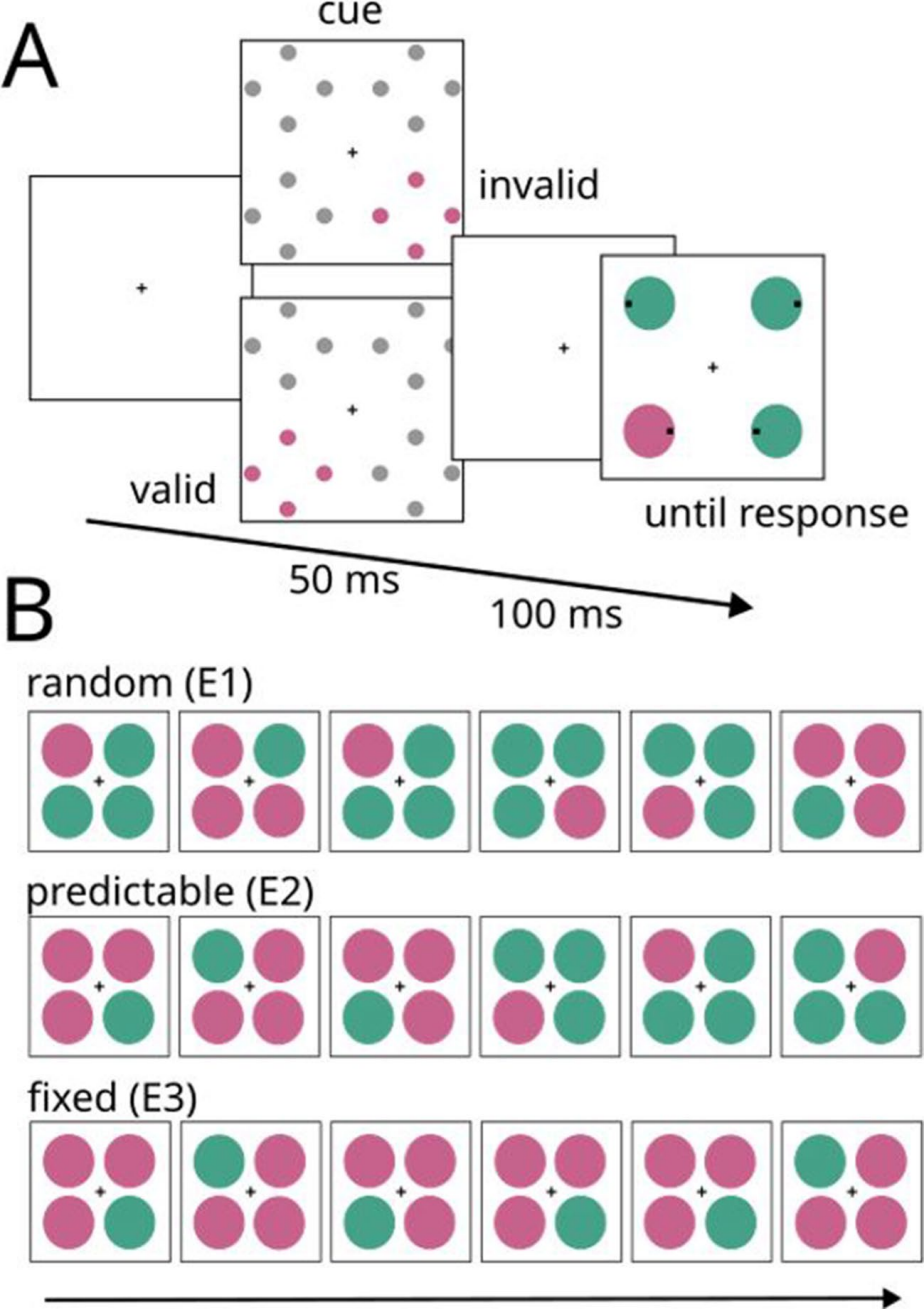
Illustration of experimental stimuli and procedure. Panel **A** illustrates the sequence of events in a trial. The cue appeared either at the target location (valid-cue trial) or at a nontarget location (invalid-cue trial). In the example, the cue has the same color as the target. Panel **B** shows sample sequences with random, predictable, and fixed colors. E1 = Experiment 1, E2 = Experiment 2, E3 = Experiment 3. (Color figure online)

In this experiment, participants searched for the color-singleton target and reported whether the black dot inside the target appeared on the left or right (see Fig. 1A). The search display was preceded by a cue display consisting of four sets of dots surrounding each of the candidate target locations. One of the sets was colored, while the others were gray. The colored set of dots is referred to as cue. The cue appeared randomly at one of the candidate target locations and either validly (25% of the trials) or invalidly (75% of the trials) cued the target location. The target and nontarget colors (red among green or green among red) as well as the cue color were randomly selected on each trial. Therefore, the cue unpredictably matched either the target or the nontarget color relative to the same trial (cue-target match vs. mismatch) and relative to the previous trial (target-cue match vs. mismatch). We conducted three separate analyses. The first two analyses were manipulation checks. First, we verified that the perceptual component of PoP could be demonstrated with the current set up. Specifically, we examined whether repetition of the target and nontarget colors on successive trials would speed performance, even when the response changed. Second, we verified that cues produced a similar cue-validity effect irrespective of the color match with the upcoming target color. The third analysis was the critical one, and its objective was to investigate whether the cue-validity effect on the current trial would be enhanced when the cue color matched the target color on the previous trial relative to when it matched the nontarget color, as predicted by the priority account of PoP.

### Methods

#### Sample-size selection and participants

The effect size of the critical effect for the present purposes—namely, the interaction between cue validity and the match between the colors of the current cue and target on the previous trial (henceforth target-cue match) in Folk and Remington (2008, Experiment 1) was *d*_z_ = 0.712. A power analysis using G*Power 3.1 (Faul et al., 2009) indicated that detecting an effect of similar size (*d*_z_ = 0.70, two-tailed, alpha = .05, power = .8) would require a sample of 18 participants. Based on this analysis, 18 students from the University of Geneva (two men; age: *M* = 20.3 years, *SD* = 1.9) participated for class credit. All reported normal or corrected-to-normal vision.

#### Apparatus

The stimuli were displayed on a 22.5-in. LCD monitor (VIEWPixx Light, VPixx Technologies Inc., Saint-Bruno, Canada). The display frequency was 100 Hz, and the pixel resolution was 1,920 × 1,200 pixels. Colors were measured with an i1Display Pro (Vpixx Edition) colorimeter by X-Rite (Grand Rapids, Michigan, USA). Head position was stabilized with a chin/forehead rest at a viewing distance of 66 cm. Responses were collected on a RESPONSEPixx Handheld 5-button response box (Vpixx Technologies Inc., Saint-Bruno, Canada), which had four buttons arranged in a diamond shape and one button in the center.

#### Stimuli

A central fixation cross (0.4° × 0.4°) was shown throughout. The search display consisted of four colored disks (2° in diameter) located at the corners of a virtual square at an eccentricity of 7.1°. Inside each disk, a small black square (0.2° in length) appeared 0.2° away from either the left or right edge of the disk. The target was either the red disk among green ones (the nontargets) or vice versa. The cue display consisted of a set of four small disks (0.6° in diameter) arranged in diamond configuration, around the location occupied by each large disk in the search display. The center-to-center distance between the large disk and each of the smaller disks was 1.4°. Three sets of small disks were light gray (xyY = 0.31, 0.33, 48.8) and one was colored (the cue), either red or green. The red and green colors were selected from an isoluminant color wheel in CIELAB space, with a lightness of L* = 59 (corresponding to a luminance of 48.8 cd/m^2^) and a saturation of 64. Distances in CIELAB space reflect perceived color distances (Fairchild, 2013). The red and green colors were on opposite sides of the color wheel at 0° and 180°. To facilitate comparison with other studies, we also indicate the colors in CIE xyY-coordinates (Y in cd/m^2^). Red was xyY = (0.44, 0.27, 48.8) and green was xyY = (0.19, 0.39, 48.8). The background was xyY = (0.312, 0.332, 24.3), which corresponds to dark gray.

#### Design

The target color was equally likely to be red or green, randomly, and therefore, the target and nontarget colors repeated or swapped randomly on consecutive trials. The cue and target positions were randomly selected, such that the cue appeared at the target location on 25% of the trials (valid trials) and elsewhere on 75% of the trials at nontarget location (invalid trials). The color of the cue was equiprobably and randomly either red or green. Therefore, on any given trial the cue was equally likely to share the color of the current target or nontargets, and also equally likely to share the color of the previous target or nontargets. The position of the small square inside the large disks was randomly selected with the constraint that in each search display, there were two squares to the right and two to the left. There were three blocks of 384 trials, resulting in 1,152 trials per participant, which took on average 37 min to complete.

#### Procedure

Each trial started with the presentation of the fixation display for 600–800 ms. Then, the cue display appeared for 50 ms, followed by the fixation display for 100 ms, which was followed by the search display. The search display remained visible until a key press was registered. Participants were instructed to search for the large disk that had a unique color and to indicate whether the small square inside the target was on the left or right by pressing the left or right key, respectively, on the RESPONSEPixx response box. Error trials as well as trials with RTs outside a 1,500-ms response window were immediately followed by visual feedback. After every 48 trials, there was a self-paced break, during which the median RT and mean error rate for these trials was displayed.

### Results

We excluded trials with RTs outside the response window (0.4%), choice errors (4%), and trials with RTs longer than 2.5 standard deviations above the respective condition mean (2%). To correct the significance of multiple t-tests, we controlled for false-discovery rate according to Benjamini and Hochberg (1995). For clarity, we report the uncorrected *p* values.

#### Target-color repetition effects

We conducted a repeated-measures 2 × 2 analysis of variance (ANOVA), with target-color repetition (repeated vs. changed) and response repetition (same vs. different) as within-subject factors.

##### Reaction times

Participants were significantly faster when the colors repeated than when they changed, 553 vs. 596 ms, *F*(1,17) = 148.72, *p* < .001, η_p_^2^ = .897. This effect was modulated by a significant interaction with response repetition, *F*(1,17) = 20.33, *p* < .001, η_p_^2^ = .545: It was larger when the response repeated than when it changed on successive trials, but crucially, it was significant both on same-response trials, 50 ms, *t*(17) = 12.05, *p* < .001, Cohen’s *d*_z_ = 2.84, and on different-response trials, 36 ms, *t*(17) = 10.08, *p* < .001, Cohen’s *d*_z_ = 2.37.

##### Accuracy

Accuracy data revealed no speed–accuracy trade-off. The main effects of target-color repetition and response repetition were significant, *F*(1,17) = 11.92, *p* = .003, η_p_^2^ = .412, and *F*(1,17) = 9.45, *p* = .007, η_p_^2^ = .357, respectively, and so was the interaction between the two variables, *F*(1,17) = 6.26, *p* = .023, η_p_^2^ = .269. Paired comparisons showed that on same-response trials, there were fewer errors when the target color repeated than when it changed, 4.1 vs. 5.7%, *t*(17) = 3.77, *p* = .002, Cohen’s *d*_z_ = 0.89, whereas there was no difference on different-response trials, 3.2 vs. 3.1%, *t*(17) = 0.23, *p* = .820, Cohen’s *d*_z_ = 0.05.

#### Influence of current cue–current target color match

We conducted a repeated-measures 2 × 2 ANOVA, with cue validity (valid vs. invalid) and cue-target color match (match vs. mismatch of the *current* cue and *current* target colors) as within-subject factors.

##### Reaction times

Participants were slower when the colors of the current cue and target matched than when they mismatched, 599 vs. 547 ms, *F*(1,17) = 63.99, *p* < .001, η_p_^2^ = .790. The interaction between cue validity and cue-target color match was significant, *F*(1,17) = 8.82, *p* = .009, η_p_^2^ = .341. It showed that the cue-validity effect was smaller with matching than with mismatching cues, −3 vs. 13 ms: paired comparisons showed that for matching cues, RTs on valid and invalid trials did not differ, 600 vs. 598 ms, *t*(17) = 0.67, *p* = .512, Cohen’s *d*_z_ = 0.16, whereas for mismatching cues, RTs were faster on valid than invalid trials, 541 vs. 554 ms, *t*(17) = 2.88, *p* = .01, Cohen’s *d*_z_ = 0.68.

##### Accuracy

Only the main effect of cue-target color match was significant, *F*(1,17) = 8.43, *p* = .010, η_p_^2^ = .331, with more errors for matching than for mismatching cues, 4.6 vs. 3.2%, respectively, mirroring the RT results.

#### Critical analysis: Influence of previous target–current cue color match

**Fig. 2:**
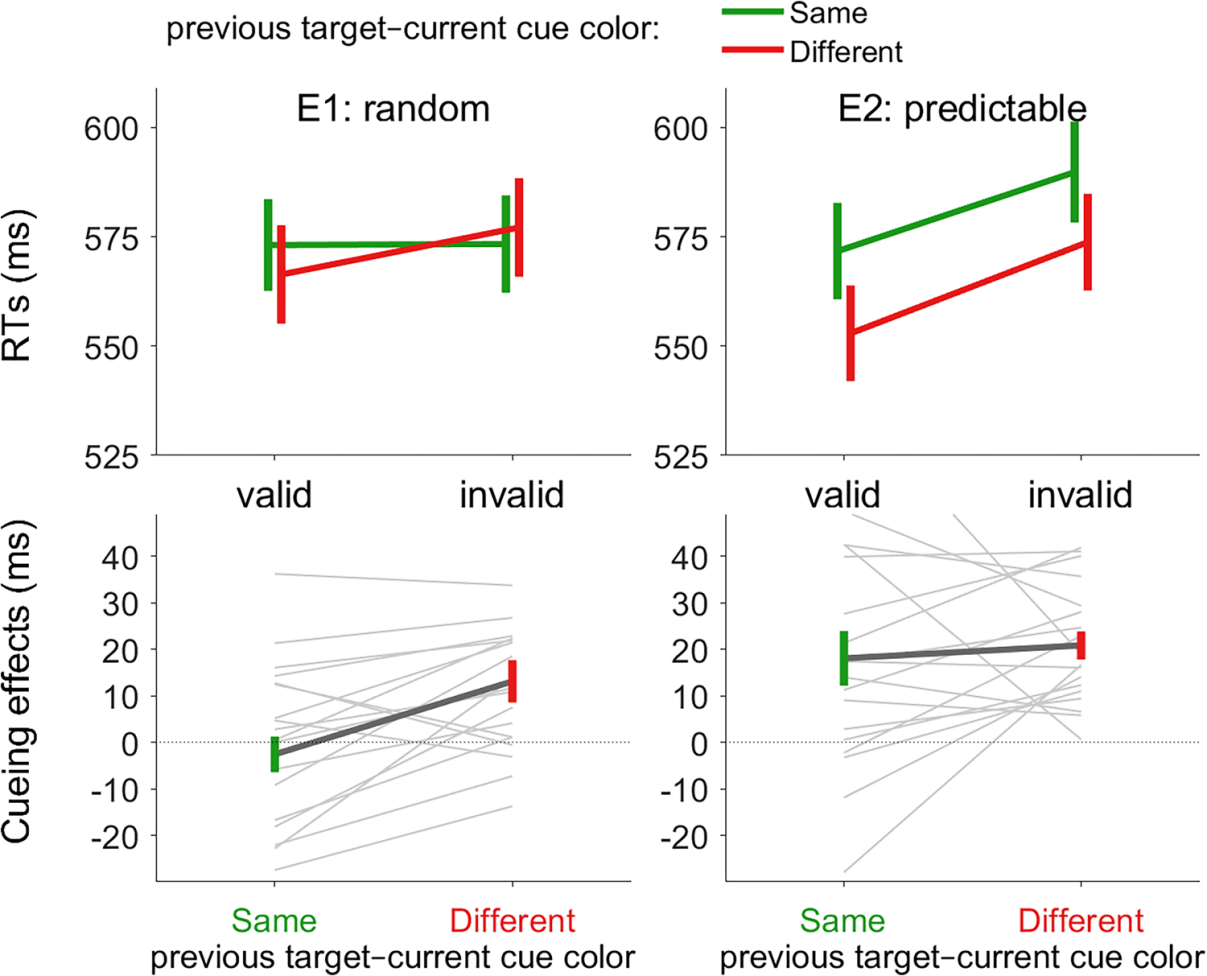
Reaction time results in Experiment 1 (random colors) and Experiment 2 (predictable colors). Data from the random- and predictable-color group are shown in the left and right panels, respectively. Absolute RTs (in ms) are shown in the top panels. Cueing effects (invalid-valid, in ms) are shown in the bottom panels. The current cue was either the same or different relative to the previous target color. Error bars show the between-participant standard error of the mean. Light-gray bars show means from individual participants. (Color figure online)

We conducted a 2 × 2 ANOVA with cue validity (valid vs. invalid) and target-cue color match (match vs. mismatch between the *previous* target color and *current* cue color) as within-subject factors (see Fig. 2).

##### Reaction times

Only the interaction between cue validity and target-cue color match was significant, *F*(1,17) = 11.05, *p* = .004, η_p_^2^ = .394. However, it indicated that the cue-validity effect was actually slightly *smaller* when the color of the current cue matched the color of the previous target than when they mismatched, 0 vs. 11 ms. That is, paired comparisons showed that RTs on valid and invalid trials did not differ for cues matching the previous target color, 573 vs. 573 ms, *t*(17) = 0.05, *p* = .958, Cohen’s *d*_z_ = 0.01, whereas RTs were faster on valid than on invalid trials for cues that did not match the previous target color, 566 vs. 577 ms, *t*(17) = 3.51, *p* = .003, Cohen’s *d*_z_ = 0.83.

##### Accuracy

Only the main effect of cue-target color match was significant, *F*(1,17) = 6.40, *p* = .022, η_p_^2^ = .273, with more errors for cues that matched the previous target color than for cues that did not, 4.3 vs. 3.6%, respectively.

### Discussion

The results of Experiment 1 are surprising on several accounts. On the one hand, we replicated the canonical PoP effect (e.g., Maljkovic & Nakayama, 1994): Search was faster when target and nontarget colors repeated than when they swapped on successive trials. Also replicating previous findings (e.g., Huang et al., 2004; Lamy et al., 2010), this PoP effect was smaller when the response changed than when it repeated but was large in both conditions. These findings are consistent with the claim that PoP has both a perceptual component and a response-based component (e.g., Lamy et al., 2010). On the other hand, the effects related to the cue were opposite to our expectations.

First, and most critically for the present purposes, a cue produced a larger cue-validity effect when its color did not match the previous target’s color than when it did. In other words, the match between the current cue and previous target colors not only did not enhance the cue-validity effect as the priority account of PoP would predict, but actually reduced it.

Second, while participants most probably searched for the odd-one-out color and both cues therefore equally matched this singleton-target set, only the cue that did not match the upcoming target color produced a cue-validity effect. In addition, this cue-validty effect was much smaller than typical cue-validity effects reported for set-matching cues in previous studies where participants were also thought to rely on singleton-detection mode to locate the target (e.g., ~50 ms; Irons et al., 2012, Experiment 1). Finally, overall performance was also better when the cue did not match the upcoming target color than when it did. This finding is inconsistent with the set-specific capture hypothesis suggested by Moore and Weissman (2010, 2011, 2014). These authors proposed that when the target unpredictably takes on one of two possible colors, detecting a distractor in one of the potential target colors automatically induces the corresponding attentional set to enter a limited-capacity focus of attention in working memory. As a result, a subsequent target is detected slower when it has the alternative color. In other words, a red cue, for instance, should have induced slower RTs for green relative to red targets. Yet, we observed the opposite pattern of results.

Taken together, the findings of Experiment 1 clearly disconfirm our prediction that moving the spatial cueing paradigm closer to Maljkovic and Nakayama’s (1994) design would enhance the impact of the previously selected target on the cuing effect. To interpret our results, however, it would be important to show that with the present set-up, the cue-validity effect can be sensitive enough to show a modulation by a variable known to affect attentional priority. The objective of Experiment 2 was to meet this goal by manipulating the observers’ attentional set.

## Experiment 2

The main objective of this experiment was to verify that the cue-validity effect was sensitive to the attentional set adopted by the observers with a procedure that differed as little as possible from that of the previous experiment. Accordingly, this experiment was similar to Experiment 1, except that instead of varying randomly, the target and nontarget colors in the search display followed a fully predictable AAABBB sequence (see Fig. 1B). Previous research has shown that participants can rapidly change their attentional set when color repetitions and swaps are fully predictable (Lien et al., 2010).^1^ Therefore, we expected the magnitude of the cue-validity effect to be larger on cue-target color match vs. mismatch trials.

As previous research has shown that PoP also occurs in fully predictable sequences (e.g., Maljkovic & Nakayama, 1994; Cochrane & Pratt, 2020), this design also allowed us to test the priority account of PoP: we could again ask whether a cue produces a larger cue-validity effect when it matches the previous target color than when it does not.

### Methods

We invited 18 participants from the same participant pool as in Experiment 1 (2 male; age: *M* = 20.4 years, *SD* = 2.0). The methods were as in Experiment 1 with the following exceptions. The target and nontarget colors varied in regular and fully predictable AAABBB sequences. Whether the color of the target in the first trial triplet was red or green was counterbalanced across participants. There were two blocks of 480 trials each, resulting in 960 trials per participant, which took on average 30 mi to complete.

### Results

We excluded trials with RTs outside the response window (0.4%), choice errors (3.1%), and trials with RTs longer than 2.5 standard deviations above the respective condition mean (2.1%).

#### Target-color repetition effects

We conducted a repeated-measures 2 × 2 ANOVA, with target-color repetition (repeated vs. changed) and response repetition (same vs. different) as within-subject factors (see Fig. 2).

##### Reaction times

The results of Experiment 1 were fully replicated. Participants were significantly faster when the colors repeated than when they changed, 555 vs. 622 ms, respectively, *F*(1,17) = 117.03, *p* < .001, η_p_^2^ = .873. This effect was modulated by a significant interaction with response repetition, *F*(1,17) = 26.17, *p* < .001, η_p_^2^ = .606: It was larger when the response repeated than when it changed on successive trials and was significant both on same-response trials, 79 ms, *t*(17) = 10.11, *p* < .001, Cohen’s *d*_z_ = 2.38, and on different-response trials, 55 ms, *t*(17) = 10.56, *p* < .001, Cohen’s *d*_z_ = 2.49.

##### Accuracy

Accuracy data revealed no speed–accuracy trade-off. The main effect of target-color repetition approached significance, *F*(1,17) = 4.03, *p* = .061, η_p_^2^ = .192, and the effect of response repetition was significant *F*(1,17) = 25.72, *p* < .001, η_p_^2^ = .602. The interaction between the two variables was also significant, *F*(1,17) = 7.18, *p* = .016, η_p_^2^ = .297. Paired comparisons showed that on same-response trials, there were fewer errors when the target color repeated than when it changed, 3.3 vs. 5.1%, *t*(17) = 2.99, *p* = .008, Cohen’s *d*_*z*_ = 0.71, whereas there was no difference on different-response trials, 2.4 vs. 2.2%, *t*(17) = 0.46, *p* = .653, Cohen’s *d*_z_ = 0.011.

#### Influence of current cue–current target color match

We conducted a repeated-measures 2 × 2 ANOVA, with cue validity (valid vs. invalid) and cue-target color match (match vs. mismatch of the *current* cue and *current* target colors) as within-subject factors.

##### Reaction times

Participants were slower when the colors of the current cue and target matched than when they mismatched, 596 vs. 549 ms, *F*(1,17) = 114.66, *p* < .001, η_p_^2^ = .871, and faster on valid than on invalid trials, 563 vs. 582 ms, *F*(1,17) = 24.59, *p* < .001, η_p_^2^ = .591. Unlike in Experiment 1, the interaction of cue validity and cue-target color match was not significant, indicating that the cue-validity effect was similar for matching and nonmatching cues, 17 ms and 20 ms, respectively, *F*(1,17) = 0.28, *p* = .607, η_p_^2^ = .016.

##### Accuracy

Only the main effect of cue-target color match approached significance, *F*(1,17) = 3.41, *p* = .082, η_p_^2^ = .167, with more errors for matching than for mismatching cues, 3.4 vs. 2.8%, respectively.

#### Critical analysis: Influence of previous target–current cue color match

We conducted a 2 × 2 ANOVA with cue validity (valid vs. invalid) and target-cue color match (match vs. mismatch between the *previous* target color and *current* cue color) as within-subject factors (see Fig. 2).

##### Reaction times

Participants were slower when the colors of the current cue and previous target matched than when they mismatched, 581 vs. 563 ms, *F*(1,17) = 52.09, *p* < .001, η_p_^2^ = .754, and faster on valid than on invalid trials, 562 vs. 582 ms, *F*(1,17) = 27.48, *p* < .001, η_p_^2^ = .618. Unlike in Experiment 1, the interaction between cue validity and cue-target color match was not significant, indicating that the cue-validity effect was similar for matching and non-matching cues, 18 ms and 21 ms, respectively, *F*(1,17) = 0.24, *p* = .630, η_p_^2^ = .014.

##### Accuracy

The ANOVA did not reveal any significant effects, *p* values > .158.

### Discussion

As in Experiment 1, while we replicated the findings relative to target repetition and its interaction with response repetition, the effects involving cue color did not conform to our expectations. Unlike in Lien et al.’s (2010) study, the cue-validity effect was similar irrespective of whether or not the cue matched the predictable color of the upcoming target. Thus, it appears that our color-predictability manipulation was not successful at inducing participants to flexibly change their attentional set to the known color of the upcoming target. However, it is noteworthy that whereas in Experiment 1, the cue-validity effect was larger when the cue color mismatched versus matched the color of the upcoming target, here, it was unaffected by cue-target color match. In other words, it seems that the strength of nonmatching cues relative to matching cues in Experiment 1 was mitigated in Experiment 2, possibly because color-predictability did in fact boost the matching cue’s priority.^2^

On top of the fact that there was no direct evidence that participants used the fully predictable color of the target to guide their search, cue-validity effects were again much smaller in this experiment (18 ms) than in previous studies where the target color was also random (e.g., Folk & Remington, 2008; Irons et al., 2012). Therefore, the results of Experiment 2 do not provide direct evidence allowing us to determine whether our paradigm was sensitive to changes in attentional priority.

## Experiment 3

One clear difference between previous studies and Experiments 1 and 2 (that motivated the present study) is that here, target and nontarget colors repeated or swapped randomly, whereas in these previous studies, the target color varied randomly but the nontargets color remained constant (and were typically gray or white). An additional potentially consequential difference is that here, the displays remained in view until response (as in most PoP experiments; e.g., Maljkovic & Nakayama, 1994), whereas in typical spatial-cueing studies (e.g., Ansorge & Heumann, 2003; Becker et al., 2013; Carmel & Lamy, 2014; Forstinger & Ansorge, 2023; Grubert & Eimer, 2016; Zivony & Lamy, 2014), including those that tested the effect of target-cue match on the cue-validity effect (e.g.,Folk & Remington, 2008; Irons et al., 2012), the search display was briefly flashed—the study by Lamy and Egeth (2003) is one exception: As here, search displays remained visible until response, and the spatial cuing effect was relatively small (around 20 ms).

We conducted Experiment 3 to determine whether we could observe large cue-validity effects with the present set-up when the colors did not swap, and whether those effects could be modulated by subtle variations of attentional priority. To do that, we used the same stimuli and procedure as in Experiments 1 and 2, except for two main changes. First, the target and nontarget colors were fixed for each participant throughout the experiment. The colors were counterbalanced across participants, and there was therefore a red-target group and a green-target group. We expected to replicate the well-established contingent-capture pattern of results (e.g., Folk et al., 1992): a large cue-validity effect for cues matching the target color (e.g., red cues when the target was also red) and a small cue-validity effect, if any, for cues that markedly differed from the target (e.g., green cues when the target was red). Second, we added a third cue color that was similar to the target color (and deviated from it by only 15°). We expected a smaller cue-validity effect for the target-similar color cue than for the target-color cue (e.g., Anderson & Folk, 2010; Kerzel, 2019).

### Methods

#### Participants

Eighteen students from the University of Geneva (five men; age: *M* = 20.2 years, *SD* = 1.4) participated for class credit.^3^ All reported normal or corrected-to-normal vision.

#### Stimuli, procedure, and design

The stimuli, procedure and design were similar to those of Experiment 1, except for the following changes. First, the target and nontarget colors remained fixed across the experiment for each participant and were counterbalanced across participants. Second, in addition to cues in the target and nontarget color, cues in a target-similar color were added, the color of which deviated from the target color by either +15° or −15°. Trials with cues in the target, nontarget, and target-similar color were equally likely and randomly mixed.

### Results

**Fig. 3:**
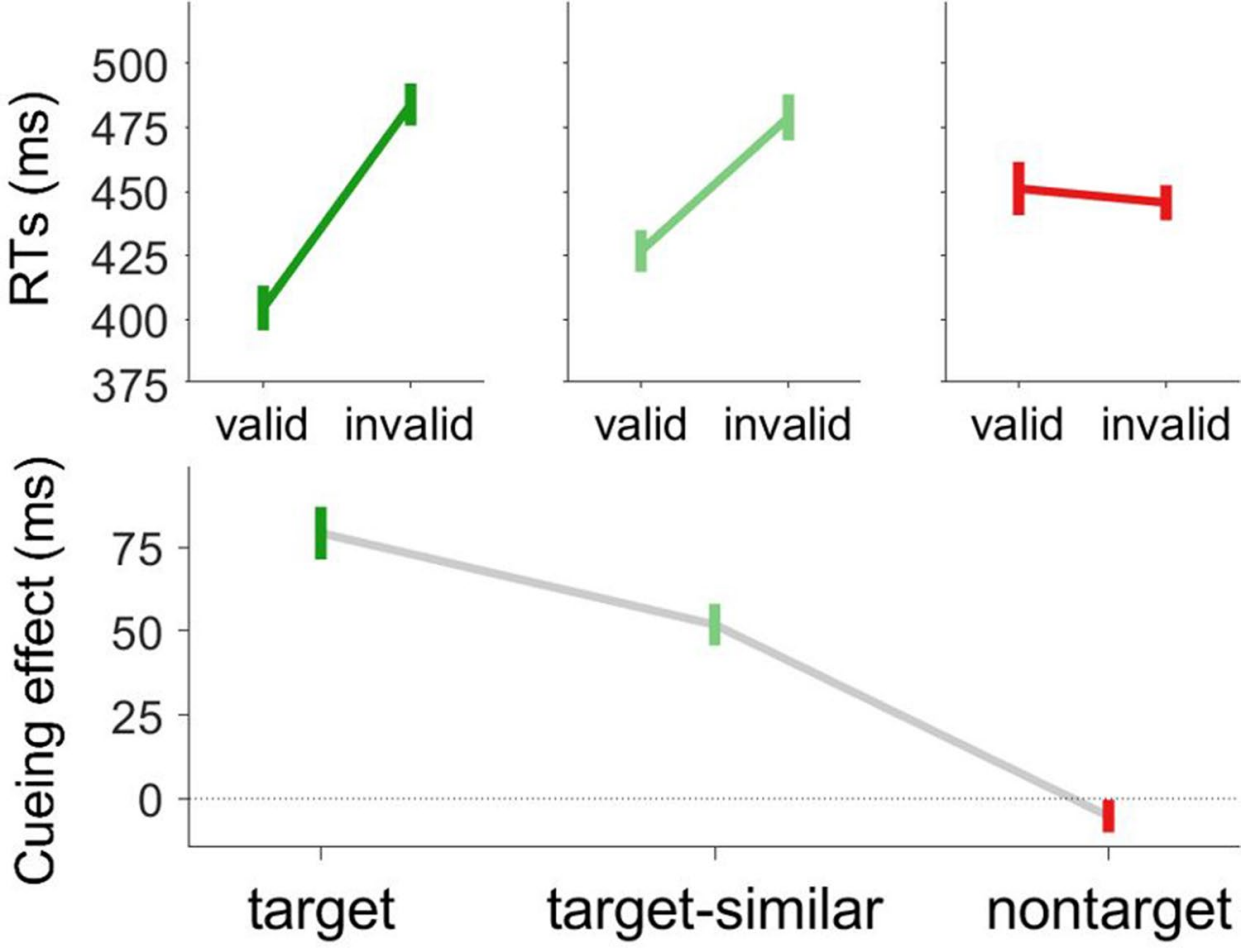
Reaction time results in Experiment 3 (fixed colors). The absolute RTs (in ms) are shown in the top panels. Note that the offset of the *y*-axis is shifted from 525 ms in Fig. 2 to 375 ms here. The cueing effects (invalid minus valid, in ms) are shown in the bottom panel. The cue was in the target color, a target-similar color, or the nontarget color. Error bars show the between-participant standard error of the mean. (Color figure online)

We excluded trials with RTs outside the response window (0.02%), choice errors (3.6%), and trials with RTs longer than 2.5 standard deviations above the respective condition mean (2.1%). The absolute RTs and cueing effects are shown in Fig. 3. We conducted a 3 × 2 ANOVA with cue color (target, target-similar, nontarget) and cue validity (valid, invalid) as within-subject variables.

#### Reaction times

The main effects of cue color and cue validity were significant, *F*(2,32) = 5.84, *p* = .007, η_p_^2^ = .267, and *F*(1,16) = 89.90, *p* < .001, η_p_^2^ = .845, respectively. Crucially, the interaction between the two factors was also significant, *F*(2,32) = 51.95, *p* < .001, η_p_^2^ = .765, indicating that cue-validity effects were larger for target than for target-similar color cues, 79 vs. 52 ms, *F*(1,17) = 15.25, *p* = .001, η_p_^2^ = .473, and for target-similar than for nontarget-color cues, 52 ms vs. −5 ms, *F*(1,17) = 48.63, *p* < .001, η_p_^2^ = 0.741. Cue-validity effects were significant for target- and target-similar color cues, *t*(17) = 10.04, *p* < .001, Cohen’s *d*_z_ = 2.37, and *t*(17) = 8.32, *p* < .001, Cohen’s *d*_z_ = 1.96, respectively, but not for nontarget-color cues, *t*(17) = 1.08, *p* = .295, Cohen’s *d*_z_ = 0.26.

#### Accuracy

The main effect of cue validity was significant, with fewer errors on valid-cue than on invalid-cue trials, 2.2% vs. 4.1%, *F*(1,16) = 13.57, *p* = .002, η_p_^2^ = .459. This effect interacted with cue color, *F*(2,32) = 7.79, *p* = .002, η_p_^2^ = .327: Mirroring the RT data, the cue-validity effect was significant for target-color cues, 3.4%, *t*(17) = 4.14, *p* = .001, Cohen’s *d*_z_ = 0.98, and for target-similar color cues, 3.0%, *t*(17) = 3.07, *p* = .007, Cohen’s *d*_z_ = 0.72, and nonsignificant for nontarget-color cues, 0.7%, *t*(17) = 0.83, *p* = .416, Cohen’s *d*_z_ = 0.35.

### Discussion

The results of Experiment 3 are straightforward. In line with the contingent-capture account, we found a large cue-validity effect when the cue matched the known-color target, and this effect was modulated by a subtle manipulation of cue-target similarity. These findings suggest that having the target and nontarget colors repeat or swap in the spatial-cueing paradigm (as was the case in Experiments 1 and 2) generates unexpected effects that mask well-replicated patterns of results with this paradigm, such as contingent capture.

## General discussion

Priming of popout is a very well-established phenomenon: in search for a singleton target, performance is faster when the target and nontarget features repeat than when they swap (see, e.g., Kristjánsson & Campana, 2010; Lamy & Kristjánsson, 2013, for reviews). Several studies examined whether this effect reflects increased attentional priority of the target feature. These studies relied on the spatial-cueing paradigm, one of the most prominent methods to study modulations of attentional priority (see Busel et al., 2020, for review). In the spatial-cueing paradigm, the cue-validity effect (i.e., the performance benefit when the target appears at the cued location vs. elsewhere) is thought to correlate with the attentional priority accruing to the cue. Consistent with this interpretation, in Experiment 3, we showed that the cue-validity effect was larger the more similar the cue was to the target: for instance, for red targets, red cues produced larger effects than pink cues and pink cues produced larger effects than green cues. Accordingly, if selecting a color increases that color’s priority, the cue-validity effect should be larger when the color of the cue on the current trial is the same as the color of the target on the previous trial. However, support for this prediction has proved elusive (see Ramgir & Lamy, for review).

Here, we tested the possibility that this inconsistent picture might result from the fact that the repetition manipulation was typically weaker in spatial-cueing studies than in PoP studies: The target feature either repeated or changed, while the nontarget feature remained constant in spatial cueing studies (e.g., Irons et al., 2012), whereas the target and nontarget features either repeated or swapped in PoP studies (e.g., McPeek et al., 1999; Wirth et al., 2023). Our findings clearly invalidated this possibility. If anything, we found a larger cue-validity effect for cues sharing the color of the previous trial’s *nontargets* than for cues sharing the color of the previous trial’s *target* (in Experiment 1).

One possible account for this finding is that on search trials with a red target among green nontargets, for instance, the larger presence of green relative to red primed the green color, and that this happened in Experiments 1 and 2 but not in Experiment 3, because the target could take on both colors in the former and only one color in the latter. However, this account is clearly post hoc. In addition, it would require further speculations to explain why such “majority-color” priming did not also emerge in the canonic target-target PoP effect replicated in Experiments 1 and 2. For instance, majority priming might occur for objects that are not associated with a task (i.e., the cue) but not for task-relevant objects (i.e., the target).

To conclude, the findings of the present study do not resolve the inconsistencies found between studies using different measures of attentional priority or using different experimental set-ups with the same measure to test the priority account of PoP. However, they allowed us to reject one clear difference between the spatial-cueing paradigm and the other paradigms (i.e., whether the target and nontarget features can swap) as a potential explanation. They also add to a growing body of research showing that spatial-cueing effects are more complex than traditionally thought and reflect a variety of processes unrelated to attention priority. For instance, it was suggested that cue-validity effects also index the speed at which attention is disengaged from the cue (Theeuwes, 2010; Theeuwes et al., 2000), the cost of updating the target’s object file when a mismatch is detected between the target’s feature and the featural information retrieved from a previous event at the same location (e.g., Carmel & Lamy, 2014, 2015), the validity of the cue on the previous trial (Goller & Ansorge, 2015; Jongen & Smulders, 2007), or the cost of encoding the cue into memory (Chen & Wyble, 2018). Here, we showed that having the target and nontarget colors repeat or swap does not only considerably reduce the cue-validity effect of cues matching the current attentional set (i.e., a set for any singleton in Experiment 1 and for a specific color in Experiment 2) but also increases the cue-validity effects generated by cues that do not match the current or previous target. While further research is needed to explain these unexpected effects, our findings highlight the need for research that directly compares the impact of well-established manipulations of attentional priority on different measures of attentional priority.

## Supplementary information

Below is the link to the electronic supplementary material.

Supplementary file1 (DOCX 209 KB)

## Data Availability

The data are available online (https://osf.io/g648j).

## Footnotes

1 We used AAABBB sequences instead of the AABB sequences used by Lien et al. (2010) as a stronger manipulation of feature-based attention.

2 However, a between-experiments analysis revealed that this difference between the two experiments was not statistically reliable: the three-way interaction between experiment, cue validity, and cue-target color match did not reach significance, *F*(1,34) = 1.70, *p* = .202, η_p_^2^ = .048. Note also that the cue-validity effect was modulated by the target-cue match in Experiment 1 and not in Experiment 2. A between-experiment analysis confirmed that the three-way interaction between experiment, cue validity, and target-cue color match was significant, *F*(1,34) = 7.34, *p* = .011, η_p_^2^ = .177. However, this difference might be due to the imbalance in the color-change probabilities between the two experiments. As noted by an anonymous reviewer, since color repetitions were twice as frequent as color changes in Experiment 2, a majority of the trials with a match between cue color and previous-target color would also have had a match between cue color and current-target color. Thus, the same presumed priority boost for cues matching the upcoming target color in this experiment relative to Experiment 1 would also account for this three-way interaction.

3 Note that these participants also performed an oculomotor capture task with similar stimuli either before or after the reported experiment, but the data from this task were discarded. Because task order was counterbalanced, we were able to evaluate whether performing the oculomotor capture task influenced performance on the cueing task. preliminary analyses showed that task order did not interact with any of the variables of interest, all *p* values > .18.

## Code availability

Requests for the program code can be sent via email to D.K.
