# Supplementary material for: Priming of pop-out in the spatial-cueing paradigm
Source: Atten Percept Psychophys. 2024 Dec 23;87(4):1150–61. doi: 10.3758/s13414-024-02998-0 (PMC12058872; doi:10.3758/s13414-024-02998-0)
Supplement: Supplementary file 1 — Supplementary file1 (DOCX 209 KB) [file 13414_2024_2998_MOESM1_ESM.docx]

**Supplementary Figure 1.**

*Choice error results in Experiments 1 and 2.*


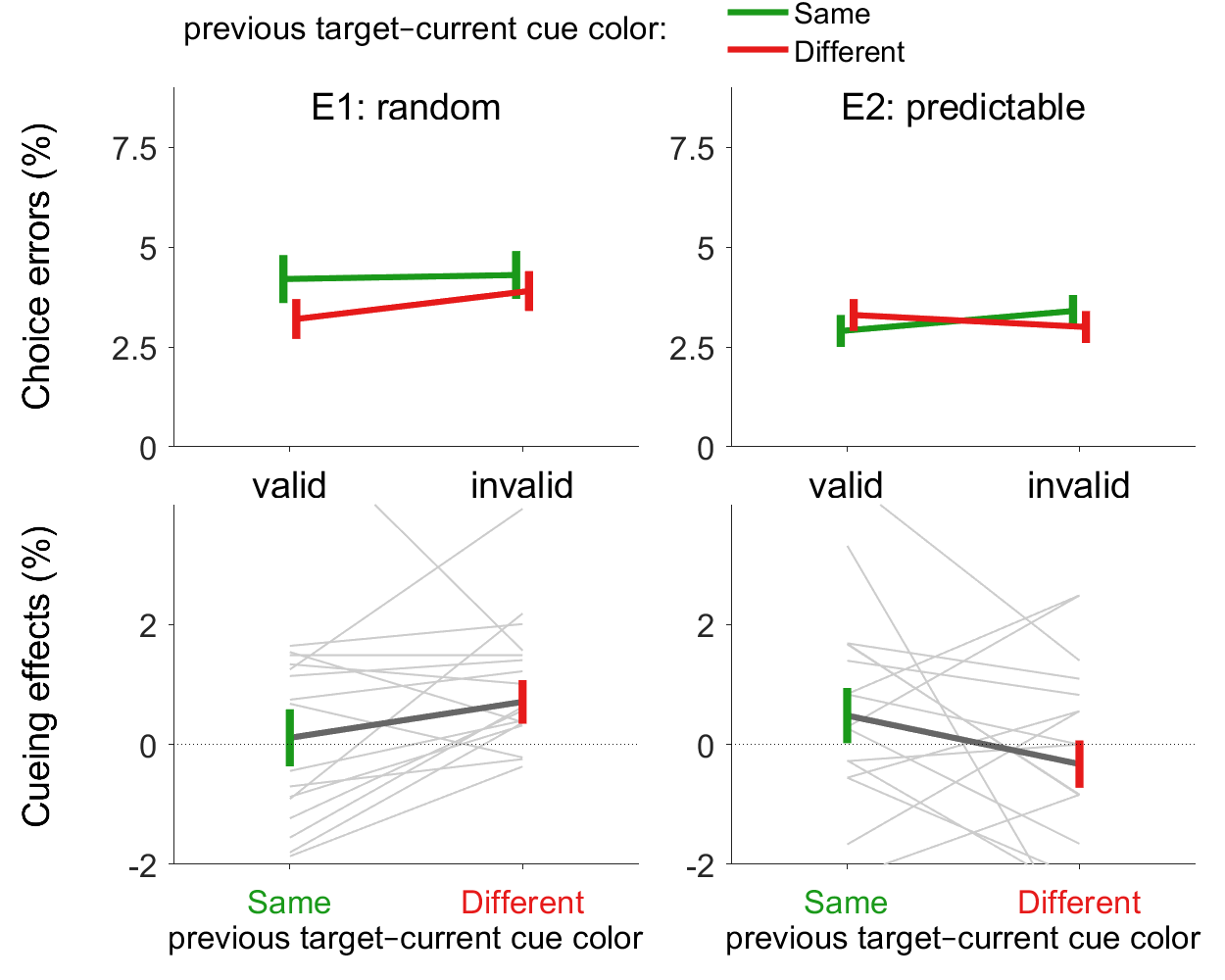


*Note.* Data from Experiments 1 and 2 are shown in the left and right panels, respectively. Choice errors (in percentages) are shown in the top panels. Cueing effects (invalid-valid, in percentages) are shown in the bottom panels. The current cue was either the same or different relative to the previous target color. Error bars show the between-participant standard error of the mean. Light gray bars show means from individual participants.
